# Therapeutic immunisation of rabbits with cottontail rabbit papillomavirus (CRPV) virus-like particles (VLP) induces regression of established papillomas

**DOI:** 10.1186/1743-422X-5-45

**Published:** 2008-03-20

**Authors:** Vandana A Govan, Edward P Rybicki, Anna-Lise Williamson

**Affiliations:** 1 Institute of Infectious Disease and Molecular Medicine, Faculty of Health Sciences, University of Cape Town, Observatory, Cape Town, South Africa; 2 Department of Molecular & Cell Biology, University of Cape Town, Observatory, Cape Town, South Africa; 3 National Health Laboratory Service, Groote Schuur Hospital, Observatory, Cape Town, South Africa

## Abstract

There is overwhelming evidence that persistent infection with high-risk human papillomaviruses (HR-HPV) is the main risk factor for invasive cancer of the cervix. Due to this global public health burden, two prophylactic HPV L1 virus-like particles (VLP) vaccines have been developed. While these vaccines have demonstrated excellent type-specific prevention of infection by the homologous vaccine types (high and low risk HPV types), no data have been reported on the therapeutic effects in people already infected with the low-risk HPV type. In this study we explored whether regression of CRPV-induced papillomas could be achieved following immunisation of out-bred New Zealand White rabbits with CRPV VLPs. Rabbits immunised with CRPV VLPs had papillomas that were significantly smaller compared to the negative control rabbit group (*P *≤ 0.05). This data demonstrates the therapeutic potential of PV VLPs in a well-understood animal model with potential important implications for human therapeutic vaccination for low-risk HPVs.

## Findings

Papillomaviruses (PVs) are small, non-enveloped viruses containing a 8 kb double-stranded closed circular DNA genome, encoding six early proteins (E1, E2, E4, E5, E6 and E7), two late proteins (L1 and L2) and a non-coding regulatory region, the long-control region (LCR) [1]. The LCR contains the origin of replication; early genes contribute to transformation and viral replication, and the late genes provide capsid proteins [1]. There are over 100 different human PV (HPV) genotypes that have been fully sequenced: the more important of these cause cervical, vulva and vaginal cancers, genital warts and recurrent respiratory papillomatosis. HPVs can be divided into low-risk, non-oncogenic or high-risk oncogenic types [2] according to their ability to cause malignant disease [3]. The most prevalent low-risk types are HPV 6 and 11, which cause 90% of genital warts (condyloma acuminata), while HPV 16 and 18 are the predominant high-risk types, causing 70% of cervical cancer and cervical intraepithelial neoplasia (CIN) [2]. Cervical cancer is the second most common cancer among women worldwide and the most common in developing countries [4] contributing significantly to a global public health burden.

In order to reduce the burden of HPV-induced infections, many studies have investigated the efficacy of different prophylactic and therapeutic vaccines in various animal models [5,6]. Preclinical studies using the cottontail rabbit papillomavirus (CRPV) in rabbits, canine oral papillomavirus (COPV) in dogs and bovine papillomavirus (BPV) in cattle have afforded a better understanding of the molecular mechanism that regulate normal cell growth, steps involved in cancerous cell changes [7] and have examined the efficacy of several delivery systems [5,8-10].

These animal studies have demonstrated that the expression of PV L1 genes in a number of cell systems results in the assembly of virus-like particles (VLPs), which elicit high titers of virus-neutralizing serum antibodies when administered as an immunogen [11,12]. As a result of the successful animal preclinical trials, L1 VLPs were effectively used as prophylactic vaccines in human clinical trials. It was demonstrated that the HPV VLP (HPV 6, 11, 16 or 18) vaccine was 100% efficacious in preventing type-specific precancerous lesions of the cervix, vulva, and vagina and effective against genital warts [13-15]. Owing to the promising human clinical trials, this prophylactic quadrivalent HPV (types 6, 11, 16 and 18) L1 recombinant VLP vaccine (Gardasil) produced by Merck was approved and registered by the Food and Drug Administration (FDA) on the 8 June 2006. Furthermore, the second preventative bivalent vaccine, Cervarix (produced by GlaxoSmithKline), which contains HPV types 16/18 L1 VLPs has been approved for use in Australia and is under review in other countries by various regulatory bodies.

However, while the current prophylactic vaccines would be effective in preventing type-specific infection it is not known whether these vaccines would afford protection to women who are already infected with the non-oncogenic HPV-associated disease. Recently, it was demonstrated that women with existing oncogenic HPV DNA infection did not benefit from HPV-16/18 L1 VLP vaccination [16]. Nevertheless, these prophylactic HPV VLP vaccines are formulated with adjuvants and are able to elicit a strong and robust immune response compared to the VLPs alone [17]. Furthermore, the immune responses elicited by the vaccine with adjuvant induced a T helper type 2 (TH2)-like response with lasting immunity compared to the VLP vaccine alone [17]. Therefore, this study used the CRPV rabbit model system, to determine whether regression of established CRPV-induced papillomas could be achieved following the vaccination of rabbits with CRPV L1 VLP vaccine, as a pilot investigation for further studies into the use of low risk HPV VLP-based vaccines as a possible therapeutic vaccine strategy.

A successful therapeutic vaccine should elicit a strong cell-mediated immune response and induce lesion regression in HPV established infection with no recurrence [18]. Indeed, studies have shown that HPV VLPs are able to induce T-cell proliferative responses in different experimental systems [19,20].

The CRPV L1 VLPs were produced in *Spodoptera frugiperda *(Sf21) cells via recombinant baculovirus as previously described [10]. Essentially, the CRPV L1 gene was directionally cloned into the pFastBac1 vector (Invitrogen), transfected into DH10Bac-competent *Escherichia coli *cells to generate bacmids which were then transfected into Sf21 cells (Invitrogen) according to the manufacturer's Bac-to-Bac protocol. The infected Sf21 cells were pelleted, resuspended in PBS containing 0.4 g/ml CsCl and complete protease inhibitor (Roche), and sonicated. The sonicated suspension was centrifuged at 100,000 g at 10°C for 24 h. Two distinct bands were observed on the CsCl gradient: the top band was extracted by puncturing the tubes and dialyzed against PBS (1.47 mM KH2PO4, 10 mMNa2HPO4, 2.7 mMKCl, 500 mMNaCl, pH 7.4) for 48 h. Dialyzed protein was divided into 100-l aliquots and frozen at 70°C for further use.

As a negative control, the rotavirus VP 6 gene, Edim (kindly provided by Dr M. Dennehy, Genbank accession number DQ019612), cloned in BCG, was used and prepared as previously described and designated pControl [9].

All animal procedures were approved by the Research Ethics Committee, Faculty of Health Sciences, University of Cape Town, South Africa. A total of nine out-bred New Zealand White rabbits (obtained from J.C rabbit Suppliers, South Africa) were infected with infectious CRPV (CRPV_Hershey _strain) at 10^-2 ^and 10^-3 ^(2 sites per dilution for each rabbit) as previously described [9]. The rabbits were randomly divided into 2 groups, and the papilloma sizes were measured 7 weeks post CRPV infection. All rabbits were immunised subcutaneously at week 8. Group 1 (n = 5) was immunized with CRPV L1 VLPs and group 2 (n = 4) was immunised with pControl 10^7 ^cfu/ml, as a negative control. Each rabbit received 3 immunisations at 2 weekly intervals. Starting at week 7 post CRPV infection the papilloma sizes were measured as length × width × height in millimetres and the geometric mean diameter (GMD) was calculated for each papilloma every week. The mean GMDs and the standard error of mean (SEM) for each group was plotted against time for sites infected with 10-2 dilution of infectious CRPV. Data were compared using the unpaired non-parametric, Mann-Whitney *U*-test. Differences were considered significant at *P *≤ 0.05.

**Figure 1:**
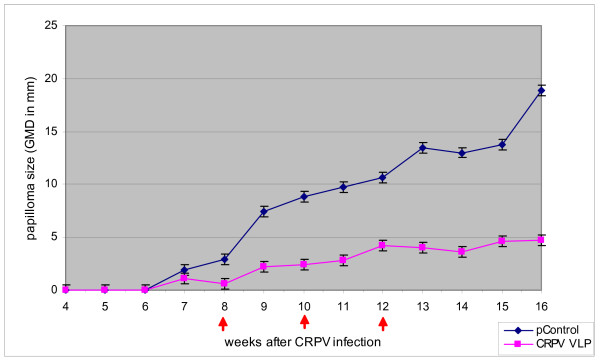
**Regression of papillomas on the backs of rabbits (NZW) following vaccination with CRPV VLPs.** A total of nine rabbits were challenged with 10-fold dilutions of infectious CRPV (10-fold dilution two sites per dilution). The rabbits were divided into two groups and immunized 3 times at 2 week intervals (↑) with CRPV VLPs(n = 5), or pControl (n = 4) antigen. The appearance of papillomas was monitored, the papilloma sizes were measured weekly beginning at week seven and the GMDs calculated. The mean GMDs and SEM of papillomas were plotted against time for the sites challenged with 10-2 dilution of infectious CRPV. *P ≤ 0.05 (Mann-Whitney *U*-test).

The rabbits in each group were monitored weekly and papilloma formation was measured each week post CRPV infection. The geometric mean diameter (GMD) for the 10^-2 ^dilution of virus (two sites per rabbit) was plotted against time after immunisation with CRPV VLPs and pControl (Fig. 1). The regression rate of the papillomas at every site for each experimental rabbit group was tabulated in Table 1. The rabbits immunised with pControl demonstrated no papilloma regression even after the third immunisation and the papillomas grew progressively. Similar progressive papilloma growths were observed in rabbits injected with PBS (pH 7.0) (data not shown). In contrast, the rabbits immunised with CRPV VLPs had papillomas that grew slower after each immunisation and were significantly smaller compared to the control group (*P *≤ 0.05) (Fig. 1). In addition, rabbits immunised with CRPV VLPs had papillomas that regressed to a GMD < 5 mm (15 of 20 sites; 75%) (Table 1) compared to no papilloma reduction in the control group.

**Table 1:** Rate of papilloma regression in each experimental rabbit group

Vaccine	Number rabbits/group	Papilloma frequency		
		
		^+^Complete regression (%)	*Rate *P *≤ value	^GMD < 5 mm (%)
CRPVVLP	5	11/20 (55)	≤ 0.05	15/20 (75)
pControl	4	0/16 (0)	-	0/16 (0)

^+^Number of papilloma sites that have completely regressed/total number of sites.

*p value; papilloma regression of each group versus control group (pControl), Mann-Whitney *U*-test.

^ Number of papilloma sites that have regressed below 5 mm, GMD/total number of sites

The results produced in this study show that immunisation with CRPV L1 VLPs is able to elicit a significant therapeutic effect in rabbits with existing CRPV induced papillomas. This is a novel result, which at first sight has significant implications for therapeutic human vaccination, given the close correspondence of the animal model to human wart pathology. However, the literature is replete with studies that show that HPV L1 VLPs generate mainly humoral responses against type-specific HPVs, and they are only effective in prophylaxis without affording a therapeutic effect against oncogenic HPV-induced infections [16,21].

The rationale for this is that the HPV life-cycle is totally intraepithelial and the virus requires a differentiated squamous epithelium to complete its life cycle and produce infectious viral particles [22]. Furthermore, in late gene expression the viral DNA is often integrated and the L1 gene would probably be disrupted. Thus the levels of L1 expression in cytotoxic T cell-accessible cells would be presumed to be undetectable, possibly preventing a L1-specific CTL response [23]. However, in a study by Zhang et al., [24] it was shown that patients with established genital warts were able to induce frequent regression when vaccinated with HPV 6b VLPs, compared to the historical controls [24]. Furthermore, comparable results were observed in a placebo-controlled human clinical trial: where women with pre-existing transient HPV 16 infection were vaccinated with HPV 16 L1 VLPs, complete protection was achieved 91.2% (95% CI, 80–97) [13]. It is suggested that the aforementioned results could be due to the secondary effects on the adjacent cells and tissues triggered by immune recognition of a primary target, also called a *bystander effect *[23]. Thus the levels of L1 would indeed be detected by the immune system but would be below the experimental (such as western blot or immunofluorescence) detection levels and would therefore be unrecognised [25,26].

Interestingly, the findings presented in the current study are in agreement with those mentioned above and we believe that the therapeutic effect afforded by CRPV L1 VLP is true. In deed, the CRPV VLP vaccine results presented here and the published commercial oncogenic HPV VLP vaccines have generated disparate results. One possible explanation for the differences observed in the CRPV model and the human trials is the route of immunisation, which could induce different immune pathways rendering different degrees of papilloma regression. Furthermore, the incidence of virus induction is dose dependant [27] and would certainly afford different papilloma regression in a natural and experimental study. In addition, although spontaneous regression of CRPV-induced papillomas have been reported to occur in less than 10% of infected rabbits [27] this was not observed in our study. The data shows that rabbits with existing CRPV-induced papillomas induced significant (*P *≤ 0.05) papilloma regression following vaccination with CRPV L1 VLPs compared to the control group which grew papillomas progressively. Thus, the results of this pilot study are surprisingly encouraging as it demonstrates for the first time in a controlled based robust animal model the therapeutic potential of the CRPV L1 VLP vaccines.

## Competing interests

The author(s) declare that they have no competing interests.

## Authors' contributions

VAG designed the study, carried out the study and drafted the manuscript. EPR provided the VLPs. A-LW participated in the coordination of the study. All authors read and approved the final manuscript.
